# Vagus Nerve Stimulation Protects Enterocyte Glycocalyx After Hemorrhagic Shock Via the Cholinergic Anti-Inflammatory Pathway

**DOI:** 10.1097/SHK.0000000000001791

**Published:** 2021-04-22

**Authors:** Juan Wu, Yushuang Yin, Mingzhe Qin, Kun Li, Fang Liu, Xiang Zhou, Xiaoyang Song, Bixi Li

**Affiliations:** ∗ Department of Anesthesiology, General Hospital of Central Theater Command of PLA, Wuhan, China; † The First School of Clinical Medicine, Southern Medical University, Guangzhou, China

**Keywords:** Electrical vagal nerve stimulation, gut barrier permeability, intestinal epithelial glycocalyx, lung injury, traumatic hemorrhagic shock/fluid resuscitation, *α7nAchR*, alpha 7 nicotinic acetylcholine receptors, *CAP*, cholinergic anti-inflammatory pathway, *EBD*, Evans Blue dye, *FD4*, fluorescein isothiocyanate dextran, *HPF*, High power field, *IL-6*, interleukin-6, *LIS*, lung injury scores, *MLA*, methyllycaconitine, *MOF*, multiple organ failure, *MPO*, myeloperoxidase, *NLR*, nucleotide-binding oligomerization domain-like receptors, *SDC-1*, syndecan-1, *THS/R*, traumatic hemorrhagic shock/fluid resuscitation, *TLR4*, Toll-like receptor 4, *TNF-α*, tumor necrosis factor-α, *VNS*, vagal nerve stimulation

## Abstract

**Introduction:**

Electrical vagal nerve stimulation is known to decrease gut permeability and alleviate gut injury caused by traumatic hemorrhagic shock. However, the specific mechanism of action remains unclear. Glycocalyx, located on the surface of the intestinal epithelium, is associated with the buildup of the intestinal barrier. Therefore, the goal of our study was to explore whether vagal nerve stimulation affects enterocyte glycocalyx, gut permeability, gut injury, and remote lung injury.

**Materials and methods:**

Male Sprague Dawley rats were anesthetized and their cervical nerves were exposed. The rats underwent traumatic hemorrhagic shock (with maintenance of mean arterial pressure of 30–35 mmHg for 60 min) with fluid resuscitation. Vagal nerve stimulation was added to two cohorts of animals before fluid resuscitation, and one of them was injected with methyllycaconitine to block the cholinergic anti-inflammatory pathway. Intestinal epithelial glycocalyx was detected using immunofluorescence. Intestinal permeability, the degree of gut and lung injury, and inflammation factors were also assessed.

**Results:**

Vagal nerve stimulation alleviated the damage to the intestinal epithelial glycocalyx and decreased intestinal permeability by 43% compared with the shock/resuscitation phase (*P* < 0.05). Methyllycaconitine partly eliminated the effects of vagal nerve stimulation on the intestinal epithelial glycocalyx (*P* < 0.05). Vagal nerve stimulation protected against traumatic hemorrhagic shock/fluid resuscitation-induced gut and lung injury, and some inflammatory factor levels in the gut and lung tissue were downregulated after vagal nerve stimulation (*P* < 0.05).

**Conclusions:**

Vagal nerve stimulation could relieve traumatic hemorrhagic shock/fluid resuscitation-induced intestinal epithelial glycocalyx damage via the cholinergic anti-inflammatory pathway.

## INTRODUCTION

Traumatic hemorrhagic shock is a common type of shock, and one of the most effective treatments is fluid resuscitation. Among patients who survive the insult of traumatic hemorrhagic shock/fluid resuscitation (THS/R), postinjury multiple organ failure (MOF), and excessive inflammation remain morbid and lethal conditions (1). Intestinal barrier dysfunction induced by THS/R might play a potential role in the development of MOFs and systematic inflammatory responses (2, 3). The process of THS/R could lead to intestinal ischemia-reperfusion injury, which disrupts the gut barrier and increases intestinal permeability. Consequently, gut epithelial cell-derived exosomes are regarded as endogenous mediators of the immune response released into the mesenteric lymph to induce proinflammatory cytokine production (4, 5). The lung is a commonly damaged distant organ, as evidenced by pulmonary inflammatory reactions, increased lung vascular permeability, and tissue edema (6).

The gut is innervated by the vagus nerve, and bidirectional communication between the brain and gastrointestinal tract is called the brain-gut axis, which plays an important role in regulating gut functions (7). The brain can modulate inflammatory responses by activating vagal nerve fibers, which is known as the cholinergic anti-inflammatory pathway (CAP). Acetylcholine released from the vagus nerve terminals combines with the alpha 7 nicotinic acetylcholine receptor (α7nAchR) on the surface of cells to regulate the levels of inflammatory cytokines (8). Vagal nerve stimulation (VNS) involves the implantation of a device to electrically stimulate the vagus nerve, which has been approved for the treatment of patients with refractory epilepsy by the Food and Drug Administration (9). Many studies have shown that VNS could prevent gut injury in THS/R-animals by activating the CAP (5, 10, 11).

Enterocyte glycocalyx is composed of transmembrane mucins, including MUC3, MUC12, and MUC17, which cover the apical surface of the enterocyte. The enterocyte glycocalyx acts as a diffusion barrier and can bind with bacteria to prevent them from passing through this barrier, whereas digested food products can pass it more easily (12–16). Many studies have shown that the dysregulation of intestinal epithelial glycocalyx is related to various human diseases, such as inflammatory bowel disease (13) and Crohn's disease (17). However, the contribution of VNS to the enterocyte glycocalyx after THS/R is unknown.

In our study, we hypothesized that the enterocyte glycocalyx could be damaged after THS/R, and VNS could protect the enterocyte glycocalyx from destruction via activating CAP, thereby decreasing gut permeability and alleviating gut and lung injury.

## MATERIALS AND METHODS

### Animals

Male Sprague Dawley rats (body weight, 250 ± 20 g) purchased from Wuhan Center for Disease Control and Prevention (Wuhan, China) were housed in cages in an environmentally controlled room at a mean temperature of 22°C with a 12/12 h light/dark cycle. Rats were allowed standard laboratory chow and tap water ad libitum. All experiments were performed in accordance with the Guide for the Care and Use of Laboratory Animals established by the US National Institutes of Health and approved by the Ethics Investigation Board of the Central Theater Command General Hospital of the Chinese People's Liberation Army, Wuhan, China.

### Model of THS/R

All rats were anesthetized with 6% chloral hydrate (5 mL/kg) intraperitoneally. After endotracheal intubation, the right carotid artery and left internal jugular vein were cannulated using PE-50 tubing. Blood pressure was monitored continuously using a BP-100 blood pressure monitor via a carotid artery catheter. Additionally, the right cervical vagus nerve was carefully exposed along the right carotid artery via the same incision. A 5 cm midline laparotomy incision was made to simulate the trauma. Blood was withdrawn through the carotid artery catheter into an anticoagulation solution syringe until the mean arterial pressure (MAP) was decreased to 30–35 mmHg and was maintained for 60 min. The rats were then resuscitated with blood and 0.9% normal saline until MAP reached 90% of baseline MAP and was maintained for 2.0 h.

### VNS

The right cervical vagus nerve was hooked with platinum electrodes, and a platinum electrode was attached to a neurostimulator. The stimulation parameters (pulse, width, frequency, and period) were set as follows: 1.0 mA, 1 Hz, 0.1 ms, and 15 min. Rats were divided into four groups (n = 6 each). Rats in the sham shock (SS) group received anesthesia and an identical procedure without hemorrhage shock, THS/R group received traumatic hemorrhage shock and fluid resuscitation; the VNS (THS/R + VNS) group received VNS before fluid resuscitation to detect the effects of VNS on THS/R rats, whereas the VNS-methyllycaconitine (THS/R + VSM) group received 10 mg/kg methyllycaconitine (MLA, Sigma–Aldrich, St. Louis, MO) (18) intraperitoneally before VNS to block α7nAchR, which mediates the anti-inflammatory effects of the efferent vagal CAP.

### Gut permeability assay

Gut permeability was determined according to descriptions of Levy (11). After resuscitation for 90 min, a 10 cm segment of the ileum was identified, incised distally, ligated proximally, and flushed with 2.0 mL of 0.9% normal saline to remove feces. Once flushed, the incision was closed. A total of 1 mL 25 mg/mL fluorescein isothiocyanate dextran (FD4, MW, 4,000 Da; Sigma, St. Louis, MO) (11) was injected into the 10 cm segment, carefully preventing any spillage onto the external bowel. After circulating for 30 min, 1 mL of venous blood was removed from the internal jugular vein into a heparinized syringe, protected from light, and placed on ice. Plasma FD4 was obtained by centrifuging at 3000 rpm for 10 min at 4°C and compared with a standard curve measured by a microplate fluorescence reader (Molecular Devices Gemini XPS, USA) at excitation 485/20, emission 528/20, and a sensitivity of 40.

### Immunofluorescence

After the rats were sacrificed, the gut tissues were collected and fixed with 4% paraformaldehyde. The tissues were dehydrated in graded alcohol and embedded in paraffin, and then the tissues were sliced into 4 μm sections. Subsequently, antigen retrieval was performed, and slices were washed with PBS three times (5 min/wash). After blocking endogenous catalase, the slices were incubated with syndecan-1 (SDC-1) primary antibody (Abcam, Cambridge, USA) and then with FITC-conjugated secondary antibody. After washing, the slices were stained with DAPI and then sealed with an anti-fade fluorescence medium. The slices were observed with a fluorescence microscope (Olympus BX53) and analyzed using ImageJ software.

### Lung vascular permeability assay

Lung vascular permeability was measured as described by Huang et al. (19). After resuscitation for 90 min, 20 mg/kg Evans Blue dye (EBD) was administered via the internal jugular vein (19). After circulating for 30 min, the lungs were perfused with 0.9% normal saline. The lungs were excised and imaged. EBD extravasation was used to assess the pulmonary vascular leakage. After imaging, the lungs were homogenized in formamide. Following overnight extraction, the tissue fluid was centrifuged at 12,000 × *g* for 10 min. The EBD concentration of the supernatant was measured at 620 nm using a microplate reader (Rayto, RT-6500, USA).

### Hematoxylin and eosin staining

Lung and gut tissues were fixed in 4% paraformaldehyde. The tissues were dehydrated in graded alcohol and embedded in paraffin, and then the tissues were sliced into 4 μm sections. Subsequently, the tissues were stained with Hematoxylin and Eosin (H&E) and observed under an optical microscope (Olympus Optical, Tokyo, Japan). The degree of lung and gut injury was assessed by an investigator blinded to the identity of the slices. Lung injury was assessed with the specific features shown in Table S1, including intravascular obstruction, inflammatory cell infiltration in the airspace or vessel wall, alveolar congestion, alveolar wall thickness, presence of amorphous material, and detachment of the bronchiole lining (20, 21). The degree of gut injury was identified by assessing intestinal mucosal damage according to the modified Chiu score, as shown in Table S2(22). Six HPFs per sample were assessed and graded based on average scores.

### Western blot analysis

The lung and gut tissues were frozen at −80°C. Proteins were extracted from lung and gut tissues using a protein extraction kit (Coolaber, China) in accordance with the manufacturer's instructions. The lung and gut tissues were homogenized on ice with RIPA buffer, and the protein concentrations were determined using a BCA protein assay kit (Coolaber, China). The protein samples were boiled in sample buffer, loaded into each lane, separated by 10% SDS-PAGE, and transferred to PVDF membranes. The membranes were washed with Tris-buffered saline with Tween 20 (TBST) three times and sealed with 5% nonfat milk for 2 h at 25°C. Subsequently, primary antibodies, including GAPDH (1:5000, ABclonal, China), MPO (1:1000, ABclonal, China), TNF-α (1:1000, ABclonal, China), IL-6 (1:1000, ABclonal, China), IL-10 (1:1000, ABclonal, China), α7nAchR (1:1000, ABclonal, China), and NF-κB p65 (1:1000, ABclonal, China) were incubated at 4°C overnight and then incubated at room temperature for 1 h. Finally, the protein levels were quantified using a chemiluminescence kit. The images were quantitatively analyzed using ImageJ analysis software.

### Quantitative real time polymerase chain reaction

Total RNA was isolated from lung tissues and gut tissues using TRIzol reagent (Simgen, China). The same amount of total RNA was reverse transcribed to cDNA using the ReverTra Ace qPCR RT kit (Simgen, China). Quantitative real time polymerase chain reaction (RT-PCR) was performed using the SYBR Premix Ex Taq (Siemens, China). The mRNA levels of target genes were detected using primers purchased from TSangon Biotech (Shanghai, China); the primer sequences are shown in Table S3. Target gene expression was quantified as the average of triplicate samples using the ^ΔΔ^CT equation and normalized to glyceraldehyde 3-phosphate dehydrogenase gene (GAPDH) expression.

### Statistical analysis

Data are presented as mean ± standard deviation (SD). One-way analysis of variance (ANOVA) with the Student–Newman–Keuls (SNK) post hoc test was used for comparisons among three groups, whereas an independent sample *t* test was used to evaluate differences between two groups. A significant difference was observed at *P* < 0.05.

## RESULTS

### THS/R destroyed enterocyte glycocalyx and VNS attenuated the damage of glycocalyx by CAP

**Fig. 1:**
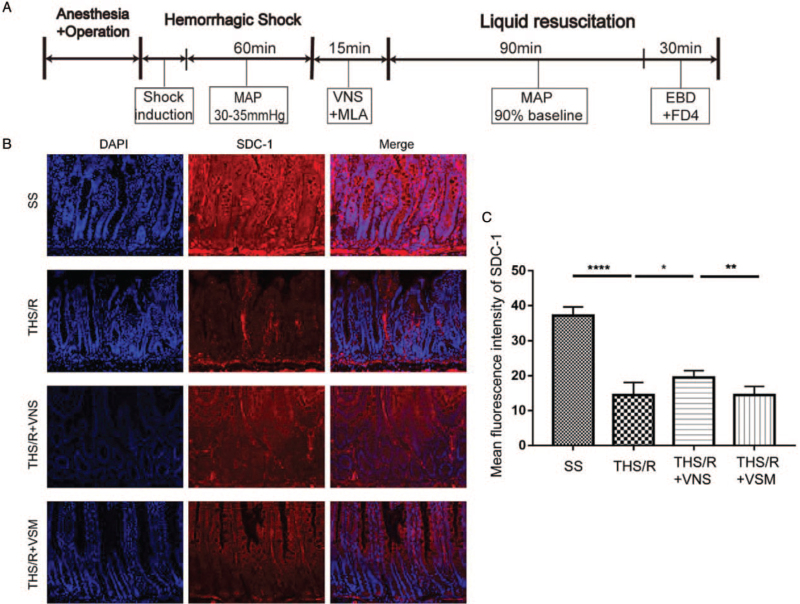
THS/R could damage the enterocyte glycocalyx and VNS could attenuate the damage of the glycocalyx by CAP. (A) The flow chart for this experiment is shown. (B) The level of SDC-1 was detected by immunofluorescence (magnification 200×) (n = 6). (C) The mean fluorescence intensity analyses of SDC-1 (n = 6). ^∗^*P* < 0.05, ^∗∗^*P* < 0.01, ^∗∗∗∗^*P* < 0.0001. CAP, cholinergic anti-inflammatory pathway; SDC-1, syndecan-1; THS/R, traumatic hemorrhagic shock/fluid resuscitation; VNS, vagal nerve stimulation.

The flow chart of this experiment is shown in Figure 1A. The content of enterocyte glycocalyx was measured using immunofluorescence (Fig. 1B). Compared with the SS group, the enterocyte glycocalyx was decreased by 61% (*P* < 0.0001). VNS caused a 35% increase in enterocyte glycocalyx compared to the THS/R group (*P* = 0.0181). Pretreatment with MAL to block CAP partly eliminated the effects of VNS (*P* = 0.0025, Fig. 1C).

### VNS decreased the inflammatory response in gut by CAP

**Fig. 2:**
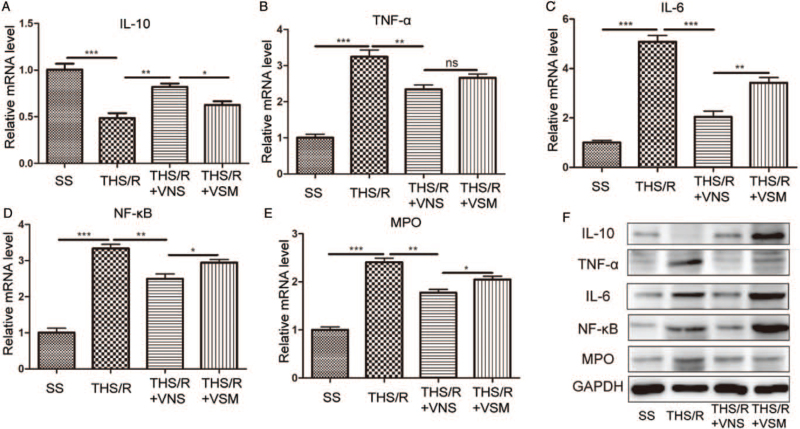
VNS decreased the inflammatory response in the gut by CAP. (A–E) IL-10, TNF-α, IL-6, NF-κB, and MPO mRNA in gut tissue were measured by RT-qPCR. (F) The expression of IL-10, TNF-α, IL-6, NF-κB, and MPO in gut tissue were measured by western-blot. ^∗^*P* < 0.05, ^∗∗^*P* < 0.01, ^∗∗∗^*P* < 0.001. CAP, cholinergic anti-inflammatory pathway; RT-qPCR, quantitative real time polymerase chain reaction; VNS, vagal nerve stimulation.

The degree of inflammatory response in the gut was reflected by the levels of inflammatory and anti-inflammatory factors, which were measured by western blotting and RT-qPCR. The α7nAchR level is important for the assessment of the anti-inflammatory effects of CAP. After administration of MLA, the α7nAchR mRNA and protein levels were downregulated via potential feedback mechanisms (23). The results showed that compared with the THS/R group, VNS upregulated the expression of α7nAchR mRNA (*P* = 0.0484). However, the expression of α7nAchR mRNA was downregulated after administration of MLA (*P* = 0.0480, Figure S1.A). The representative blots showed that the variations in the α7nAchR protein levels were similar to the changes in mRNA levels (Figure S1. B). Compared to the SS group, the mRNA level of IL-10 was downregulated in the THS/R group (*P* = 0.0007). The mRNA level of IL-10 was upregulated in the THS/R + VNS group compared to that in the THS/R group (*P* = 0.0018). However, compared with the THS/R + VNS group, IL-10 mRNA levels were downregulated in the THS/R + VSM group (*P* = 0.0105). Compared to the SS group, the mRNA levels of TNF-α, IL-6, NF-κB, and MPO were upregulated in the THS/R group (TNF-α, *P* < 0.0001; IL-6, *P* < 0.0001; NF-κB, *P* < 0.0001; MPO, *P* < 0.0001, Fig. 2A). The mRNA levels of TNF-α, IL-6, NF-κB, and MPO were downregulated following treatment with VNS for 15 min (TNF-α, *P* = 0.0070; IL-6, *P* = 0.0001; NF-κB, *P* = 0.0033; MPO, *P* = 0.001). Subsequently, IL-6, NF-κB, and MPO mRNA levels were upregulated in the THS/R + VSM group (IL-6, *P* = 0.0045; NF-κB, *P* = 0.0305; MPO, *P* = 0.0326, Fig. 2C–E). However, TNF-α mRNA levels were not different between the THS/R + VNS and THS/R + VSM groups (*P* = 0.1027, Fig. 2B). The representative blots showed that the variations in these protein levels were similar to the changes in mRNA levels (Fig. 2F).

### VNS reduced gut histology injury and decreased gut permeability by CAP

**Fig. 3:**
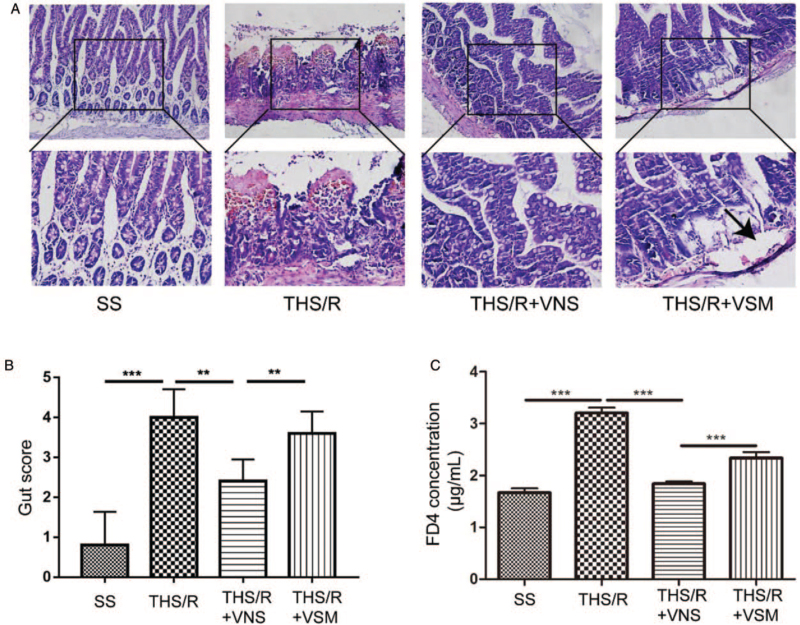
VNS reduced histological gut injury and decreased gut permeability by CAP. (A) Histopathological examination of intestine by H&E staining. (B) The concentration (μg/mL) of FD4 in the plasma was measured with a microplate fluorescence reader at excitation 485/20 nm, emission 528/20 nm, with a sensitivity of 40. ^∗∗∗^*P* < 0.001. CAP, cholinergic anti-inflammatory pathway; FD4, fluorescein isothiocyanate dextran; H&E, hematoxylin and eosin; VNS, vagal nerve stimulation.

After ischemia/reperfusion injury, the gut tissues exhibit subepithelial space, moderate lifting of the epithelial layer from the lamina propria, massive epithelial lifting down the sides of the villi, denuded villi with lamina propria, and dilated capillaries. Gut permeability was evaluated by measuring the plasma FD4 levels. Compared with the SS group, gut injury was characterized by the lifting of the epithelial layer from the lamina propria and denuded villi with lamina propria in the THS/R group (*P* = 0.0002). After treatment with VNS, gut injury was alleviated compared with the THS/R group (*P* = 0.0039). However, the gut injury was aggravated after administration of MLA in the THS/R + VSM group, exhibiting subepithelial space (*P* = 0.0085, Fig. 3A and B). Compared with the SS group, gut permeability increased by 200% in the THS/R group (*P* < 0.0001). Gut permeability decreased by 50% in the THS/R + VNS group compared with that in the THS/R group (*P* < 0.0001). However, compared with the THS/R + VNS group, gut permeability increased by 21% in the THS/R + VSM group (*P* = 0.0007, Fig. 3C).

### VNS decreased pulmonary vascular permeability and alleviated lung histological damage by CAP

**Fig. 4:**
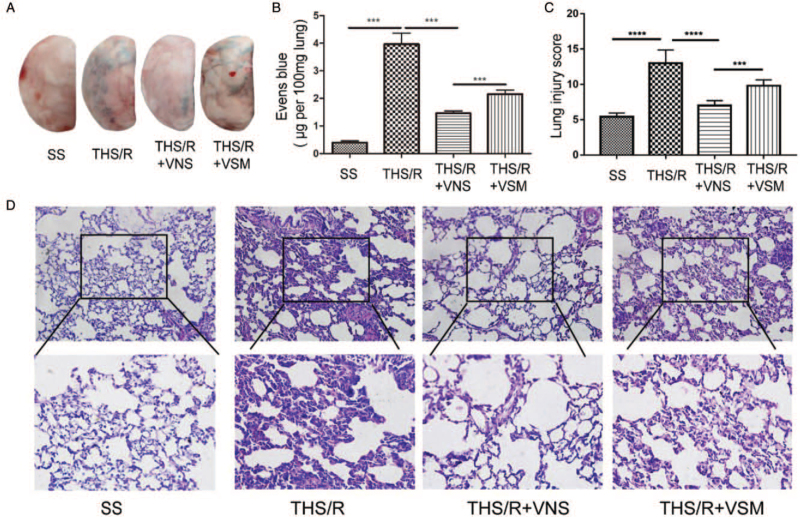
VNS decreased pulmonary vascular permeability and alleviated histological lung damage by CAP. (A) Images of lung tissue are shown. (B) The EBD level was measured with a microplate reader at 620 nm absorption. (C) The LIS of lung tissues are shown. (D) Histopathological examination of lung by H&E staining. ^∗∗∗^*P* < 0.001, ^∗∗∗∗^*P* < 0.0001.CAP, cholinergic anti-inflammatory pathway; EBD, Evans Blue dye; LIS, lung injury scores; VNS, vagal nerve stimulation.

Lung injury induced by THS/R is characterized by increased pulmonary vascular permeability, inflammatory cell infiltration, and pulmonary edema; lung injury scores (LIS) are used to quantify lung injury. Pulmonary vascular permeability was evaluated by measuring the degree of EBD extravasation. Compared with the SS group, THS/R increased pulmonary vascular permeability 12-fold (*P* < 0.0001). After treatment with VNS, pulmonary vascular permeability decreased by 63% (*P* < 0.0001). However, compared with the THS/R + VNS group, pulmonary vascular permeability increased by 32% in the THS/R + VSM group (*P* = 0.0009, Fig. 4A and B). Furthermore, compared with the SS group, lung tissues were dramatically damaged in the THS/R group, showing inflammatory cell infiltration in the airspace and increased alveolar wall thickness (*P* < 0.0001). Lung injury was alleviated after treatment with VNS compared with that in the THS/R group (*P* = 0.0002). However, compared with the THS/R + VNS group, pretreatment with MLA aggravated lung injury in the THS/R + VSM group (*P* = 0.0004, Fig. 4C and D). These results are similar to those of previous studies (5, 10, 11).

### VNS reduced the inflammatory response in lung tissue by CAP

**Fig. 5:**
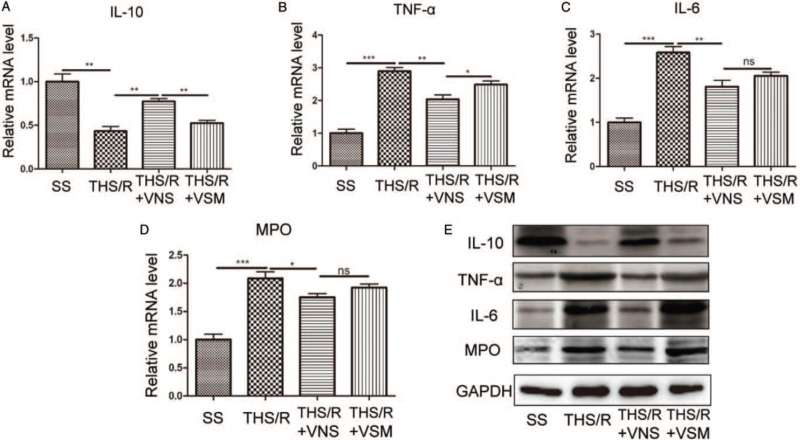
VNS reduces the inflammatory response in lung tissue by CAP. (A–D) IL-10, TNF-α, IL-6, and MPO mRNA in lung tissue were measured by RT-qPCR. (E) The expression of IL-10, TNF-α, IL-6, and MPO in lung tissue was measured by western-blot. *ns*, non-significant; ^∗^*P* < 0.05, ^∗∗^*P* < 0.01, ^∗∗∗^*P* < 0.001. CAP, cholinergic anti-inflammatory pathway; RT-qPCR, quantitative real time polymerase chain reaction; VNS, vagal nerve stimulation.

IL-10, TNF-α, and IL-6 are biomarkers of inflammatory response in the lung, and the MPO level reflects neutrophil activation in the lung, which plays an important role in the development of lung injury (24). The mRNA and protein levels were measured by RT-qPCR and western blotting, respectively. Compared to the SS group, the mRNA level of IL-10 was downregulated in the THS/R group (*P* = 0.0015). The mRNA level of IL-10 was upregulated in the THS/R + VNS group compared with that in the THS/R group (*P* = 0.0015). However, IL-10 mRNA levels were downregulated in the THS/R + VSM group by blocking CAP compared with the THS/R + VNS group (*P* = 0.0014, Fig. 5A). Compared with the SS group, the mRNA levels of TNF-α, IL-6, and MPO were upregulated in the THS/R group (TNF-α, *P* < 0.0001; IL-6, *P* < 0.0001; MPO, *P* = 0.0003). After treatment with VNS, the mRNA levels of TNF-α, IL-6, and MPO were downregulated in the THS/R + VNS group compared with those in the THS/R group (TNF-α, *P* = 0.0031; IL-6, *P* = 0.0074; MPO, *P* = 0.0441). The mRNA level of TNF-α was upregulated by blocking CAP compared with that in the THS/R + VNS group (*P* = 0.0453, Fig. 5B). However, the IL-6 and MPO mRNA levels were not different between the THS/R + VNS and THS/R + VSM groups (IL-6, *P* = 0.1735; MPO, *P* = 0.1002, Fig. 5C and D). The representative blots showed that the variations in these protein levels were similar to the changes in mRNA levels (Fig. 5E). These results are consistent with those of previous studies (5, 10, 11).

## DISCUSSION

As one of the leading causes of morbidity and mortality in working-age people worldwide, THS/R could result in a systematic inflammatory response, eventually leading to disability or mortality (25–27). Recent studies have demonstrated that gut injury induced by THS/R plays a crucial role in the development of the above procedures, indicating that THS/R could damage the gut barrier and increase intestinal barrier permeability. Next, gut epithelial cell-derived exosomes, regarded as endogenous mediators of the immune response, are released into the mesenteric lymph to induce a proinflammatory cytokine production (4, 5). Fishman et al. (28) revealed that the intraluminal unstirred mucus layer is involved in the gut injury induced by THS/R. The mucosal layer, gut microbiota, intestinal immune system, and integrity of intestinal epithelial cells are important components of the intestinal barrier. The enterocyte glycocalyx is composed of transmembrane mucins, including MUC3, MUC12, and MUC17, and covers the apical surface of the enterocyte (12). Numerous studies have shown that enterocyte glycocalyx acting as a diffusion barrier could maintain a physical barrier and bind with bacteria to prevent them from passing this barrier (29). Furthermore, the dysregulation of enterocyte glycocalyx could contribute to human diseases, such as inflammatory bowel disease (17) and colorectal cancer (30). In this study, THS/R damaged enterocyte glycocalyx, increased gut barrier permeability, and caused gut and lung injury.

Goblet cells dispersed among the intestinal epithelial cells are responsible for the synthesis and secretion of transmembrane mucins, which could be influenced by interactions with the immune system (31). By induction of the NF-κB family of transcription factors, many pattern recognition receptors, such as TLRs and NLRs, play essential roles in mucin synthesis (32). Hasnain et al. (33) indicated that high IL-10 levels could prevent mucin misfolding. Additionally, some cytokines, including IL-1, IL-6, and TNF-α, and acetylcholine released from the vagus nerve, have important effects on mucin synthesis (34–36). In our study, THS/R increased IL-6 and TNF-α levels and decreased the expression of IL-10 in the gut, resulting in marked damage to the enterocyte glycocalyx. After administration of VNS, IL-6, and TNF-α levels were downregulated and IL-10 levels were upregulated, and enterocyte glycocalyx damage was relieved.

The gut is innervated by the vagal nerve, and CAP is a neuroimmunomodulation mechanism that regulates inflammation of the central nervous system and vagal nerve. In this paradigm, the immune system can activate afferent fibers of the vagal nerve to transmit the stimulus signals to the central nervous system, which integrates the stimulus signals. In turn, the central nervous system transmits the integrated signals to the vagal nerve terminal by activating the efferent vagal fibers. Then the vagal nerve terminal releases acetylcholine to combine with α7nAchR on the surface of inflammatory cells to regulate inflammatory cytokine levels by activating the Jak2-STAT3 signaling pathway and inhibiting the NF-κB pathway (8, 37). α7nAchR mediated the efferent vagal anti-inflammatory effects of CAP. Giebelen et al. (38) demonstrated that activating CAP could not alleviate the inflammatory response in α7nAchR knockdown animal models.

VNS might be an effective method to alleviate gut injury and remote organ injury, including lung injury. There are many explanations for the protective effects of VNS. Many studies have demonstrated that VNS could alleviate gut barrier damage and gut injury, as well as decrease the inflammatory factor levels following THS/R. However, pretreatment with α7nAchR blockers could reverse the protective effects of VNS, suggesting that the protective effects of VNS might be mediated by CAP activation (10, 39, 40). Recently, Coimbra et al. (41, 42) suggested that gut epithelial cell-derived exosomes secreted into mesenteric lymph were relevant to systematic inflammation response after THS/R by activating monocyte NF-κB and increasing macrophage TNF-α production. Additionally, Coimbra et al. (42) defined the role of these exosomes as critical mediators of THS/R-induced lung injury through macrophage TLR4 activation. Subsequently, William et al. (43) demonstrated that VNS could relieve the systematic inflammation response by attenuating the transition of mesenteric lymph exosomes to the THS/R-induced inflammatory phenotype. In this study, VNS decreased the levels of IL-6, TNF-α, and NF-κB and increased IL-10 levels in gut tissues, thereby alleviating the damage of the enterocyte glycocalyx. However, when we pretreated with MLA to block α7nAchR, the protective effects of VNS were reversed, suggesting that VNS could inhibit the inflammatory response in the gut and relieve enterocyte glycocalyx damage after THS/R via CAP. Combined with the study by Raul Coimbra et al. (41, 42), the relationship between the enterocyte glycocalyx and the production of mesenteric lymph exosomes requires further study.

Acute lung injury, characterized by increased pulmonary vascular permeability, inflammatory cell infiltration, and pulmonary edema, is a common complication in patients after THS/R. Previous studies have shown that increased gut permeability plays an important role in the development of lung injury during THS/R. Specifically, gut-derived cytokines and mesenteric lymph exosomes were carried into the superior vena cava via the mesenteric lymphatic system. After the right ventricle contracts, the lung is the first organ exposed to these cytokines and exosomes, which results in inflammatory responses in lung tissues (25). In this study, THS/R increased gut permeability, damaged intestinal mucosa, caused inflammatory cell infiltration in lung tissues, and increased pulmonary vascular permeability. After administration of VNS, the gut mucosal damage and lung injury were relieved and pulmonary vascular permeability decreased. However, when we pretreated with MLA to block α7nAchR, the protective effects of VNS were reversed, suggesting that the protective effects of VNS were mediated by CAP. These findings are consistent with those of previous studies (5, 10, 11).

In conclusion, we demonstrated that the enterocyte glycocalyx was damaged after THS/R, and VNS might relieve intestinal epithelial glycocalyx damage induced by THS/R via CAP, which provides a new insight into the protective effects of VNS. Although this study made advances based on previous research, there were some limitations. First, the effects of VNS may activate or block vagal afferent and/or efferent vagal activity; thus, further studies are necessary to determine the specific VNS effects in our study. Second, MLA is not a specific α7nAchR antagonist and may act at a higher level than nicotinic acetylcholine receptors. Additionally, the sample size was inadequate for our study. Third, the effects of VNS on upstream genes of the enterocyte glycocalyx require further study.

## Supplementary Material

Supplemental Digital Content

## Supplementary Material

Supplemental Digital Content

## Supplementary Material

Supplemental Digital Content

## Supplementary Material

Supplemental Digital Content
