# Supplementary material for: Vagus Nerve Stimulation Protects Enterocyte Glycocalyx After Hemorrhagic Shock Via the Cholinergic Anti-Inflammatory Pathway
Source: Shock. 2021 Apr 22;56(5):832–9. doi: 10.1097/SHK.0000000000001791 (PMC8519159; doi:10.1097/SHK.0000000000001791)
Supplement: Supplemental Digital Content [file shk-56-832-s001.docx]

Table S1 Lung histology scoring system

| Table S1 Lung histology scoring system | | | |
| --- | --- | --- | --- |
| Score | **Vascular features** | **Extravascular and alveolar involvement** | **Bronchiole features** |
| 0 | Minimal | Minimal | None |
| 1 | Blood leaking into interstitium;  mild RBC obstruction | Mild inflammatory exudate; areas of patchy oedema with some disordered structure | Mild infiltration of inflammatory cells |
| 2 | Mild RBC and vascular  obstruction; areas of mild and moderate haemorrhage | Moderate inflammatory exudate; areas of moderate  alveolar thickening (25–50% visualized lung) | Moderate infiltration of inflammatory cells;  detachment of lining in some bronchioles |
| 3 | Diffuse haemorrhage;  moderate RBC and vascular obstruction | Moderate-severe inflammatory exudate; Severe alveolar thickening (> 50% visualized lung); loss of structure with amorphous material | Complete loss of bronchiole structure;  detachment of lining; cellular debris and  inflammatory cell exudate |

*RBC* red blood cell
