# Supplementary material for: Vagus Nerve Stimulation Protects Enterocyte Glycocalyx After Hemorrhagic Shock Via the Cholinergic Anti-Inflammatory Pathway
Source: Shock. 2021 Apr 22;56(5):832–9. doi: 10.1097/SHK.0000000000001791 (PMC8519159; doi:10.1097/SHK.0000000000001791)
Supplement: Supplemental Digital Content [file shk-56-832-s002.docx]

Table S2 Modified Chiu scoring system

| Table S2 Modified Chiu scoring system | |
| --- | --- |
| Grade | **Features of mucosa** |
| 0 | Normal mucosa histomorphology |
| 1 | Slight separation of epithelial cells from the lamina propria at the tip of the villus. A subepithelial space has formed at the tip of the villus (“Gruenhagen’s space”) |
| 2 | Extension of the subepithelial space and minimal demarcation between epithelial cells and lamina propria with partial loss of the epithelial cells at the tip of the villus |
| 3 | The epithelial separation from the lamina propria has progressed from the tip towards to the base, exposing one-third to one-half of the lamina propria, moderate vasodilation and congested capillaries in lamina propria and tela submucosa |
| 4 | Nearly complete to complete loss of epithelium with marked vasodilation, congested capillaries und hemorrhage in the lamina propria and edema in the tela submucosa |
| 5 | Complete loss of villus architecture leaving an irregular denuded surface, disintegration of the lamina propria, necrosis of the crypts |
