# Supplementary material for: Vagus Nerve Stimulation Protects Enterocyte Glycocalyx After Hemorrhagic Shock Via the Cholinergic Anti-Inflammatory Pathway
Source: Shock. 2021 Apr 22;56(5):832–9. doi: 10.1097/SHK.0000000000001791 (PMC8519159; doi:10.1097/SHK.0000000000001791)
Supplement: Supplemental Digital Content [file shk-56-832-s003.docx]

Table S3. Primer sequences

| Table S3 Primer sequence in this study | | |
| --- | --- | --- |
| Gene name | Forward (5’→3’) | Reversed (5’→3’) |
| MPO | TCGTATTTCAAGCAGCCGGT | GCATGTCCCCTGTGACATTGAA |
| TNF-α  IL-6  IL-10  NF-κB p65  α7nAchR | AACTCGAGTGACAAGCCCGTG  ACTTCCATCCAGTTGCCTTCTT  AAGGGTTACTTGGGTTGCCA  TACCACTGTCAACAGATGGCCC  AGGCTGTACAAGGAGCTGGT | GTACCACCAGTTGGTTGTCTTTGA  TCATTTCCACGATTTCCCAGA GTGTCACGTAGGCTTCTATGC  ATATGCCGTCCTCACAGTGC  TGATCTGCAGGAGACTCAGG |
| GAPDH | GGCACAGTCAAGGCTGAGAATG | ATGGTGGTGAAGACGCCAGTA |
