# Supplementary material for: Vagus Nerve Stimulation Protects Enterocyte Glycocalyx After Hemorrhagic Shock Via the Cholinergic Anti-Inflammatory Pathway
Source: Shock. 2021 Apr 22;56(5):832–9. doi: 10.1097/SHK.0000000000001791 (PMC8519159; doi:10.1097/SHK.0000000000001791)
Supplement: Supplemental Digital Content [file shk-56-832-s004.docx]

Figure S1


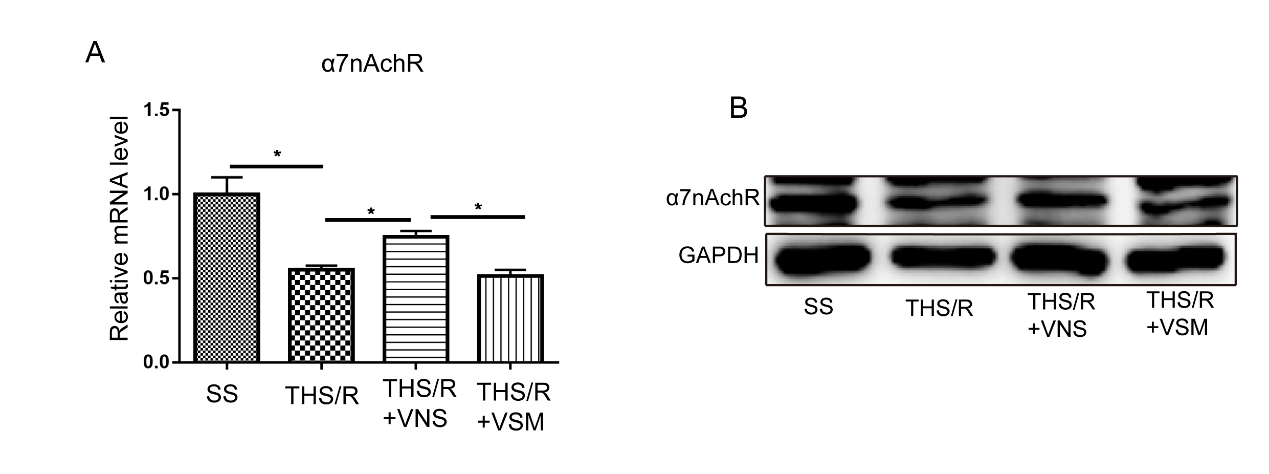


**Figure.S1**. The effect of VNS on α7nAchR mRNA and protein level in gut. (A) The mRNA of α7nAchR in gut was measured by RT-qPCR. (B) The protein of α7nAchR in gut was measured by Western blot. Data are shown as the mean ± SD (n=6). *p<0.05.
